# Alert Reduction and Telemonitoring Process Optimization for Improving Efficiency in Remote Patient Monitoring Programs: Framework Development Study

**DOI:** 10.2196/66066

**Published:** 2025-06-13

**Authors:** Job van Steenkiste, Niki Lupgens, Martijn Kool, Daan Dohmen, Iris Verberk-Jonkers

**Affiliations:** 1 Faculty of Management Sciences, Open University, Valkenburgerweg 177, Heerlen, 6419 AT, The Netherlands, 31 455762888; 2 Department of Internal Medicine, Maasstad Hospital, Rotterdam, The Netherlands; 3 Department of Hospital Pharmacy, Erasmus MC University Medical Center, Rotterdam, The Netherlands; 4 Luscii Healthtech BV, Utrecht, The Netherlands

**Keywords:** care delivery, remote patient monitoring, remote monitoring, patient monitoring, telemonitoring, alert, alerts, workflow, workflows, hypertension monitoring, hypertension, high blood pressure, medical informatics, data, data processing, algorithm, algorithms, model, models, analytics

## Abstract

**Background:**

Telemonitoring can enhance the efficiency of health care delivery by enabling risk stratification, thereby allowing health care professionals to focus on high-risk patients. Additionally, it reduces the need for physical care. In contrast, telemonitoring programs require a significant time investment for implementation and alert processing. A structured method for telemonitoring process optimization is lacking.

**Objective:**

We propose a framework for optimizing efficient care delivery in telemonitoring programs based on alert data analysis and scenario analysis of a telemonitoring program for hypertension combined with a narrative literature review on methods to improve efficient telemonitoring care delivery.

**Methods:**

We extracted 1-year alert processing data from the telemonitoring platform and electronic health records (June 2022-May 2023) from all users participating in the hypertension telemonitoring program in the outpatient clinic of the Department of Internal Medicine of the Maasstad Hospital. We analyzed the alert burden and alert processing data. Additionally, a scenario analysis with different threshold values was conducted for existing blood pressure alerts to assess the impact of threshold adjustments on the overall alert burden and processing. We searched for English language academic research papers and conference abstracts reporting clinical alert or workflow optimization in telemonitoring programs on May 24, 2024 in Embase, Medline, Cochrane, Web of Science, and Google Scholar.

**Results:**

In total, 174 users were included and analyzed. On average, each user was active in the telemonitoring program for 207 days and a total of 30,184 measurements were performed. These triggered a total of 17,293 simple, complex, and inactive or overdue alerts: 13,647 were processed automatically by the telemonitoring platform, and 3646 were processed manually by e-nurses from the telemonitoring center, equivalent to 21 manually processed alerts per user. Additional analysis of the manually processed alerts revealed that 25 (15%) users triggered more than 50% of these specific alerts. Furthermore, scenario analysis of the alert thresholds revealed that a single increase of 5 and 10 mmHg for the diastolic and systolic blood pressure alerts would reduce the number of alerts by about 50%, resulting in a total reduced time investment for the e-nurse of 5973 minutes over 1 year. Literature search yielded 251 articles, of which 7 studies reported methods to improve efficiency in telemonitoring programs, including the introduction of complex alerts and clinical algorithms to triage alerts, scenario analysis with alert threshold adjustments, and a qualitative analysis to create an alert triage algorithm.

**Conclusions:**

Based on the data analysis and literature review, a 4-step framework was developed to optimize the efficiency of telemonitoring programs. The 4 steps include ensuring accurate measurements, telemonitoring algorithm and alert optimization, focusing on individual users’ and user groups’ needs, and improving telemonitoring process efficiency. This framework can be an important first step to improve the efficiency of 21st-century telemonitoring programs.

## Introduction

Since the COVID-19 pandemic [1], the use of telemonitoring has rapidly increased [2]. In 21st-century telemonitoring, patients measure relevant health data like vital signs at home according to a predefined measurement schedule [3]. These data are transmitted through a smartphone or tablet application, can be reviewed remotely by health care providers, and can trigger alerts based on pre-defined threshold values. Alerts are reviewed by e-nurses in remote patient monitoring centers and discussed with health care providers if required. Depending on the alert, remote treatment adjustment will take place, and feedback is provided to the patient. In theory, the application of telemonitoring improves health care delivery by contributing to the Quintuple Aim goals [4] aimed at improving patient experiences, improving population health, reducing health care costs, improving care team well-being, and enhancing health equity. Achieving specific efficiency-related goals requires telemonitoring to reduce the need for physical care and shift attention to patients who mostly require it. This is particularly relevant in situations with increased care needs combined with reduced available health care staff [5]. It is however, for many diseases still unclear whether telemonitoring does lead to more efficient care delivery, as it requires a significant time investment (eg, alert processing, development and implementation of telemonitoring algorithms), generates new data, and needs continuous optimization of clinical workflows [6,7].

Modern telemonitoring platforms are often embedded in existing care paths and include clinical algorithms that triage and process the generated alerts [3,8]. This proactive way of telemonitoring ensures that patients who are off target are quickly identified, and actions can be undertaken proactively to prevent deterioration of the patient’s condition. Conversely, stable patients do not generate alerts and thus do not require immediate care.

These health data [8] ideally generate as few alerts as possible, and a clinical consequence is attached to each generated alert. However, in practice, alert triggering and processing are not perfect yet. Algorithms that have strict alert thresholds generate many alerts, increase clinical workload, and enhance so-called “alert fatigue” [9], whereas algorithms with very wide alert thresholds generate few alerts but may pose significant health risks, for example, by underreporting relevant changes in a patient’s health status (“do not harm principle”) [5]. “Alert fatigue” is an important barrier when developing a telemonitoring algorithm [10]. In hypertension management, for example, it is known that health care providers, on average, only respond to around 60% of generated off-target blood pressure (BP) alerts [11]. To improve the efficiency of a telemonitoring program, technical improvements or algorithm enhancement, such as designing more sophisticated alerts or refining threshold values, can be initiated [12,13]. However, a clear, structured method like the use of a framework to reduce alerts and improve telemonitoring efficiency is lacking.

In this study, we evaluate the efficiency of a large telemonitoring program for hypertension at the Maasstad Hospital in Rotterdam, the Netherlands, by analyzing telemonitoring alerts and alert processing. Additionally, we use scenario analysis to assess the impact of adjusting alert thresholds on the overall alert burden. Finally, we combine this analysis with a narrative literature review on methods to improve telemonitoring efficiency to propose a framework for alert reduction and telemonitoring process optimization.

## Methods

### Study Design

This study used a cross-sectional data analysis, narrative literature review, and subsequential framework development.

### Setting

This study was conducted at the Maasstad Hospital, a large teaching hospital in Rotterdam, the Netherlands.

### Ethical Considerations

The study was conducted following the principles of the Declaration of Helsinki Version: WMA 52nd General Meeting, Edinburgh, Scotland, Oct 2000 (Washington 2002 and Tokyo 2004) [14] and Good Clinical Practice standards [15]. This study was approved by the Institutional Review Board of the Maasstad Hospital (identifier L2024-029). This study was assessed by the Medical Ethics Committee United and not subject to the Medical Research Involving Human Subjects (identifier W24.053). As all data were processed anonymously, a consent procedure was waived by both the Institutional Review Board and the Medical Ethics Committee United.

### Telemonitoring Organization and Algorithm

**Figure 1.**
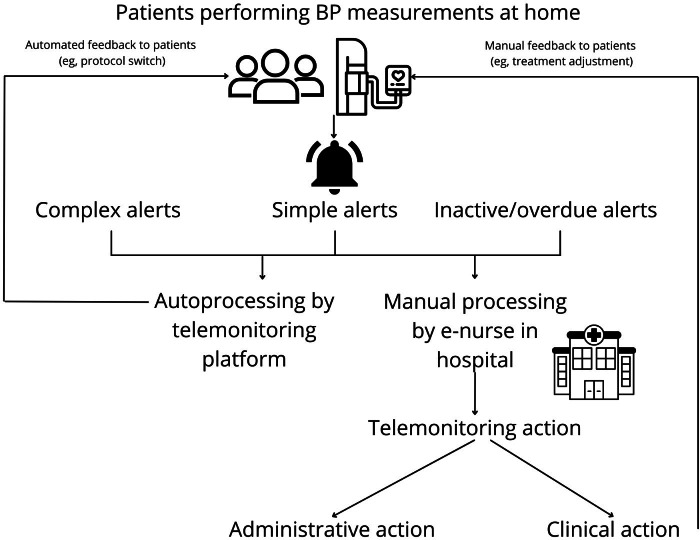
Telemonitoring organization and alert processing. Users measure their blood pressure at home using a validated Bluetooth blood pressure machine. These measurements can trigger simple, complex, or inactive or overdue alerts. These alerts are either processed automatically by the telemonitoring platform or manually by the e-nurse in the hospital. Alerts are processed on a single day for a single user during a telemonitoring action. This can either be an administrative or clinical action. Feedback to the users is provided automatically by the telemonitoring platform (eg, a protocol switch) or manually by the e-nurse (eg, a treatment adjustment).

The Maasstad Hospital has extensive experience with telemonitoring for various medical conditions. Since June 2022, a central telemonitoring center has been in operation where specialized monitoring nurses assess all generated alerts during office hours. They are supervised daily by a responsible nurse specialist or medical specialist. See Figure 1 for a graphical overview of the telemonitoring organization.

### Hypertension Care Path and Telemonitoring Program

The hypertension program and telemonitoring application (Luscii) [16] used in this study have been available since 2021 and are embedded in a care path for hypertension. The program is developed in line with the latest European Society of Cardiology/European Society of Hypertension guidelines [17]. Measurements are performed using a Bluetooth-connected, validated BP monitor. The Luscii application consists of a smartphone—or tablet application and an web-based healthcare provider dashboard. BP data are transmitted directly to the health care provider dashboard via the smartphone or tablet application. Once transmitted to the health care provider dashboard, the BP data are analyzed based on predefined algorithms, which will subsequently generate alerts (see details below under “Telemonitoring Alerts and Alert Processing”) which are only visible in the health care provider dashboard. In the user application, users can review their BP data and have access to relevant educational modules and self-care documents, which, for example, emphasize the need for smoking cessation and antihypertensive drug adherence.

Users measure their BP twice in the morning and twice in the evening during measurement weeks. The program consists of 4 acute measurement algorithms and 1 chronic measurement algorithm. The acute algorithms, with BP targets ranging from 180/110 to 150/95 mmHg, have higher frequencies of measurement weeks and stricter threshold values and are used during the initial titration phase of achieving BP control. We choose 180/110 mmHg as the highest initial target during the titration phase, as higher BP can be associated with hypertensive emergencies [17]. Stable patients with on-target BP measurements are included in the chronic algorithm (140/90 mmHg) and measure 1 week per month. Patients automatically move between algorithms when BP values meet the target thresholds during the measurement weeks. See Multimedia Appendix 1 for a complete overview of the measurement algorithms.

### Home Blood Pressure Telemonitoring Program Users

The hypertension care path is limited to an outpatient hospital setting, and general practitioners are currently not involved. All patients with hypertension, treated in the department of internal medicine of the Maasstad Hospital, either primarily for hypertension or, for example, when hypertension is a relevant risk factor (eg, diabetes, chronic kidney disease), are offered to participate in this digital care path. Exclusion criteria are the existence of communication problems, lack of sufficient digital skills, and the absence of the possession of a mobile device. Validated BP measurement equipment can be loaned from the hospital if patients are unable to afford the purchase of such measurement. Once included, around 70% of the total care provided will be conducted remotely and digitally and 30% of the follow-up consultations will still be provided in person. The patients who primarily require follow-up for hypertension are referred by a general practitioner due to suspected secondary hypertension, resistant hypertension, drug intolerances, or a suspected hypertensive emergency. Given the heterogeneity of this group and for the purpose of this study, which focuses on organizational and efficiency aspects of telemonitoring, we refer to this complete patient group as the “users” of the telemonitoring program.

### Telemonitoring Alerts and Alert Processing

The monitoring program generates 3 different types of alerts (Figure 1) following BP data transmission by the users of the telemonitoring program. First, there are simple alerts, which are triggered by a 1-time measurement exceeding predefined thresholds, eg, a very high or low systolic or diastolic BP. Second are complex alerts, which are triggered if a series of measurements are off target (eg, multiple consecutive off-target BP measurements) or if a combination of measurements (eg, systolic and diastolic BP and heart rate) are off target. In practice, these alerts are used for clinical decisions, such as drug treatment adjustments. Third are alerts that are triggered when users either miss a single scheduled measurement (overdue alert) or when they have missed all measurements within x days (inactive alert). Alerts can be processed automatically (eg, a single overdue alert, or an alert when a user is “on target”) by the telemonitoring platform while triggering automated workflows created by the responsible clinicians. These workflows usually contain automated feedback messages that are delivered to the user automatically when the alert is processed by the monitoring platform (eg, your BP has improved, your target has changed). Besides autoprocessing by the telemonitoring platform, alerts can also be processed manually (eg, a complex alert for off-target BP) by e-nurses in a telemonitoring center. Whether alerts need manual processing by the e-nurses depends on choices made by health care providers in the monitoring algorithm. Simple alert thresholds were based on expert opinion, as no guidelines for these specific alerts are available. Thresholds for the complex alerts were based on treatment targets for home BP monitoring in existing guidelines [17]. We specifically choose to make the alert thresholds slightly higher than the treatment targets to reduce the overall alert burden (eg, 140/90 compared to 135/85). All alerts generated on a single day by a single user are clustered and manually processed using a telemonitoring action by the e-nurse, consisting of either an administrative action or a clinical action (eg, a treatment adjustment). Administrative actions have no clinical impact, as these actions only involve administrative handling of the alert in the telemonitoring platform and electronic health record (EHR) without any clinical consequences (eg, no treatment adjustments). Prior to processing the alerts, e-nurses can decide to contact patients to gather relevant clinical information (eg, adherence to antihypertensive drugs of lifestyle interventions). They can also provide feedback to the patients, for example, following antihypertensive treatment adjustment. Patients will also be contacted following inactivity alerts. Contacting patients is performed via a digital secured messaging platform (BeterDichtbij [18]) or via a telephone consultation. Alerts that need manual processing generated on Saturdays and Sundays were combined into one action on Monday, as the e-nurses only review alerts during office hours.

### Telemonitoring Data

We extracted 1-year alert and alert processing data from the Luscii telemonitoring platform [16] and EHRs from all users participating in the hypertension telemonitoring program between June 1, 2022 and the May 31, 2023. Given the focus of the study, only anonymized data and no clinical or demographic data were extracted.

### Alert Analysis

Alerts were processed either automatically or manually. We assigned each alert to a specific measurement to assess the impact of various alert thresholds on the number of generated alerts. Manually processed alerts clustered in telemonitoring actions were categorized, resulting in an administrative or a clinical action. Clinical actions can be a telephone consultation (with or without a treatment adjustment) or an integrated secured asynchronous message (with or without a treatment adjustment) through a communication platform.

### Literature Search

For the narrative literature review, we searched articles reporting clinical alerts or workflow optimization in telemonitoring programs on May 24, 2024 in Embase, Medline, Cochrane, Web of Science, and Google Scholar. The complete search strategy is included in Multimedia Appendix 2. We included all English language academic research papers and conference abstracts published after January 1946. Studies reporting on alert reduction in clinical, in-hospital, settings (eg, intensive care units) were excluded. Relevant literature was identified using title and abstract screening from the initial identified papers. Relevant studies were subsequently reviewed in full text, and relevant alert reduction methods or workflow optimizations were extracted by JvS.

### Analysis

Data from the telemonitoring platform and EHR were analyzed using Microsoft Excel. Alert and alert processing data were reported as frequencies (N) and proportions (%). A separate scenario analysis with different threshold values was conducted for all simple systolic and diastolic alerts for all included users that were processed manually by the telemonitoring center. The number of alerts that would be triggered with the new threshold values was based on the absolute values of the measurements performed in the original dataset. We calculated the current percentage of alerts that were processed administratively and extrapolated these to the alerts with the adjusted threshold values to estimate the percentage of alerts that would be processed administratively after the threshold adaptations and give insight into the potential workload reduction for the e-nurses. In this scenario analysis, the assumption was made that any alert generated by the telemonitoring program that would be processed administratively would not have any clinical impact on the user.

### Framework Development

We created a framework for alert reduction and clinical workflow optimization in telemonitoring programs based on an iterative process that included a synthesis of our own clinical experiences, the alert-reduction and clinical workflow optimization methods identified in our own study (eg, scenario analysis), the methods for alert reduction as identified in the narrative alert reduction literature review, and general recommendations available from other relevant telemonitoring studies.

## Results

### Telemonitoring Alerts

**Figure 2.**
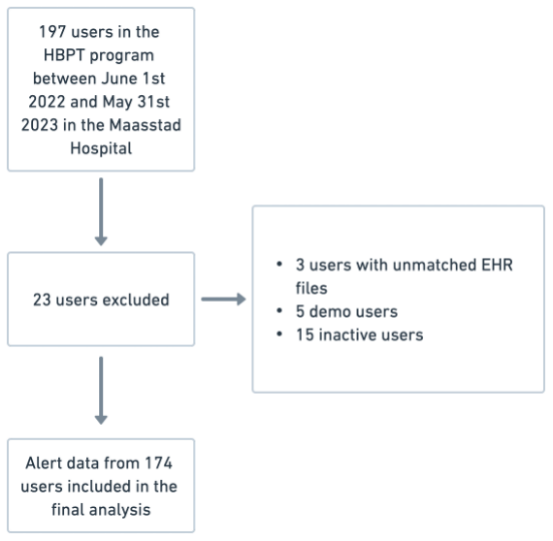
Flow diagram illustrating the telemonitoring data collection.

Between June 1, 2022, and May 31, 2023, 197 users were included in the home blood pressure telemonitoring program. 5 users were identified as demo users, 3 users could not be matched to an EHR file, and 15 users did not activate the telemonitoring app. Therefore, alert data from a total of 174 users were included in the final analysis (Figure 2). In total, 45% of these users started in the chronic (monthly) measurement algorithm and 55% in the acute (weekly or biweekly) measurement algorithms. On average, each user was active in the telemonitoring app for 207 days during the study period, and a total of 30,184 measurements were performed. These measurements triggered 17,293 alerts, of which 13,647 were autoprocessed and 3646 were processed manually, equivalent to 21 manually processed alerts per user (Table 1).

**Table 1.** Telemonitoring alert data between June 1, 2022 and May 31, 2023.

Variable	Value
Total number of active users	174
Average days active	207
Total number of measurements	30,184
Total number of alerts (% of total number of measurements)	17,293 (57)
Number of alerts auto processed (% of total number of alerts)	13,647 (79)
Complex alerts (% of total number of alerts autoprocessed)	1878 (14)
Inactivity or overdue alerts (% of total number of alerts autoprocessed)	11,769 (86)
Number of alerts processed manually (% of total number of alerts)	3646 (21)
Simple alerts (% of total number of alerts processed manually)	2025 (56)
Complex alerts (% of total number of alerts processed manually)	1344 (37)
Inactivity or overdue alerts (% of total number of alerts processed manually)	277 (7)

Of the autoprocessed alerts, 11,769 (86%) were overdue alerts and 1878 (14%) were complex alerts. Of the manually processed alerts, 2025 (56%) were simple, 1344 (37%) were complex, and 277 (7%) were inactivity alerts. The 277 manually processed overdue or inactivity alerts were generated by 73 (42%) of all included users. It is important to note that overdue alerts are triggered when a single measurement is not completed in time. Within a hypertension protocol consisting of multiple scheduled measurements per day, this can have a large impact on the total number of alerts. However, due to the automated processing of these alerts, their contribution to the clinical workload is negligible.

### Telemonitoring Center Data

The 3646 alerts that were processed manually accounted for a total of 2101 monitoring actions (all alerts that were processed manually on a single day for 1 user clustered into 1 action) and 1626 (77%) of these actions were processed with an administrative action (Table 2). A total of 475 (23%) alerts resulted in a clinical action. Out of these, 191 (40%) were telephone consultations without a treatment adjustment, 130 (28%) were telephone consultations with a treatment adjustment, 134 (28%) were digital messages without a treatment adjustment, and 20 (4%) were digital messages with a treatment adjustment. On average, 1 monitoring action for each user per month was registered based on the total average participation of 207 days (7 months). In total, the combined average workload for the e-nurses was 6.2 minutes per user per month, assuming a workload of 5 minutes for administrative actions and 10 minutes for clinical actions (telephone or messaging).

**Table 2.** Telemonitoring center data between June 1, 2022 and May 31, 2023.

Variable	Value
Total telemonitoring actions^a^	2101
Administrative actions (% of total number of monitoring actions)	1626 (77)
Clinical actions (% of total monitoring number of actions)	475 (23)
Telephone consultations without treatment adjustment (% of total number of clinical actions)	191 (40)
Telephone consultations with treatment adjustment (% of total number of clinical actions)	130 (28)
Digital messages without treatment adjustment (% of total number of clinical actions)	134 (28)
Digital messages with treatment adjustment (% of total number of clinical actions)	20 (4)

a All alerts that were generated on a single day were combined into 1 “telemonitoring action” in which the e-nurses reviewed all generated alerts on 1 specific day for 1 single user.

In total, 25 (15%) users triggered 61% of the overall simple alerts and 48% of the complex alerts that were processed manually. These users were responsible for 45% of all monitoring actions, which meant that the e-nurses would roughly spend half of their time on a very small subgroup (15%) of the included users.

### Adjusting Individual Algorithm Thresholds

Review of the telemonitoring actions of the 25 users who generated most alerts led to individual adaptations in 13/25 users. In 3/13 users, the alert thresholds could be adjusted based on medical decisions (eg, accepting higher BP due to perceived intolerance of antihypertensive drugs, accepting asymptomatic lower heart rate), which would result in a reduction of 265 manually processed simple alerts in these users. Additionally, 3/13 users were provided with additional measurement instructions, and 7/13 users were discharged from the telemonitoring program due to a lack of digital skills or inability to adhere to the required measurement schedule and continued with regular physical care.

### Adjusting Group Algorithm Thresholds: Scenario Analysis

A scenario analysis (Table 3) was conducted with various adaptations in thresholds for the 2025 simple systolic and diastolic alerts that were processed manually. We found that increasing the threshold values by only 5 mmHg for the simple diastolic alert and only 10 mmHg for the simple systolic alert would reduce the total number of alerts by 47% and 54%, respectively. These adjustments would lead to small increases in required clinical actions of 4% and 3%, respectively, if the alert processing remains similar to the current threshold scenario. Therefore, this scenario revealed limited clinical implications despite a large reduction in overall alerts. This was similarly applicable for adjusting the diastolic threshold value to ≥115 mmHg and the systolic threshold value to ≥190 mmHg, as in these scenarios, the increase of alerts requiring a clinical action was around 11% and 8%, respectively.

**Table 3.** Scenario analysis for the manually processed simple diastolic and systolic alerts between June 1, 2022 and May 31, 2023.

Alert type	% currently processed with administrative action/clinical action (total number of alerts)	Current threshold	New threshold	% total reduction in alerts (number of alerts)	% processed with administrative action or clinical action after threshold adjustment (total number of alerts) (% difference)	Potential time saving in minutes for the e-nurses
Diastolic simple alert	71 (726)/29 (297)	≥105 mmHg	≥110 mmHg	47 (481)	67 (322)/33 (159) (−4/+4)	2993
Diastolic simple alert	71 (726)/29 (297)	≥105 mmHg	≥115 mmHg	77 (788)	60 (473)/40 (315) (−11/+11)	4951
Diastolic simple alert	71 (726)/29 (297)	≥105 mmHg	≥120 mmHg	91 (930)	50 (465)/50 (465) (−21/+21)	5908
Systolic simple alert	71 (620)/29 (253)	≥170 mmHg	≥180 mmHg	54 (471)	68 (320)/32 (151). (−3/+3)	2980
Systolic simple alert	71 (620)/29 (253)	≥170 mmHg	≥190 mmHg	85 (742)	63 (467)/37 (275) (−8/+8)	4734
Systolic simple alert	71 (620)/29 (253)	≥170 mmHg	≥200 mmHg	95 (829)	44 (365)/56 (464). (−27/+27)	5290

We calculated potential time savings for the e-nurses based on the 2025 simple alerts in the current threshold scenario that were processed manually. These consisted of 1023 diastolic alerts, 873 systolic alerts, and 129 heart rate alerts. In total, 71% (726 and 620) of the 1023 diastolic and 873 systolic simple alerts were processed with administrative actions and 29% (297 and 253) with clinical actions, which would lead to a total workload for the e-nurses of 6598 minutes for the diastolic alerts and 5631 minutes for the systolic alerts, assuming a 5-minute workload for each individual administrative action and a 10-minute workload for each individual clinical action. In the adjusted threshold scenarios, we calculated total time savings based on a reduced workload due to the total reduction of alerts and an increased workload due to an increased percentage or alerts that would require a clinical action. We assumed a 10-minute workload for a clinical action. The potential time savings for the e-nurses were 2993, 4951, and 5908 minutes for the diastolic simple alerts and 2980, 4734, and 5290 minutes for the systolic alerts, respectively, assuming a 10-minute workload for each individual clinical action.

### Literature Review

The original search yielded 251 articles. Based on the predefined exclusion criteria, 241 articles were excluded as they did not report any alert reduction methods or describe studies in intensive care units or other in-hospital settings. In total, 10 articles were reviewed in full text, and 7 of these articles contained a clear description of a method to improve telemonitoring efficiency. These methods included the introduction of complex alerts to reduce the overall alert burden, the introduction of clinical algorithms to triage alerts, scenario analysis with alert threshold adjustment, and a qualitative analysis to create an alert triage algorithm. See Table 4 for a complete overview of the studies reporting methods to improve telemonitoring efficiency.

**Table 4.** Overview of studies reporting methods to improve telemonitoring efficiency.

Author	Year	Type of telemonitoring program	Described method to improve telemonitoring efficiency
Vuković et al [Bibr R13][13]	2010	Heart failure	Introduction of complex alerts
Cuba Gyllensten et al [Bibr R22][19]	2017	Heart failure	Introduction of complex alerts Introduction of clinical algorithms to triage alerts
Vamos et al [Bibr R23][20]	2018	Heart failure	Introduction of complex alerts
Zahradka et al [Bibr R24][21]	2022	General vital signs	Scenario-analysis with alert threshold adjustment to reduce alert burden
Nguyen et al [Bibr R25][22]	2023	Elderly care	Qualitative analysis of health care provider notes to create an alert triage algorithm
Richman et al [Bibr R26][23]	2023	Cardiac implantable devices	Scenario-analysis with alert threshold adjustment to reduce alert burden
Mazza et al [24]	2024	Oncology	Introduction of clinical algorithms to triage alerts

## Discussion

### Principal Findings

Based on our alert data analysis and narrative literature review, we identified multiple areas to reduce alerts and enhance telemonitoring efficiency. First, by using automated workflows in the telemonitoring program, around 86% of alerts could be processed automatically. Reduction of the alerts could also be achieved by individual adjustment of the alert thresholds: review of the subset of users who generated most of the workload, both in terms of the generated alerts as well as the workload burden for the e-nurses, posed opportunities to make individual threshold adjustments. These findings highlight the need for a more individualized approach with personal thresholds and measurement schedules in certain cases. Additionally, in our scenario analysis, we showed a large potential in alert reduction and time saving for the e-nurses for the simple alerts with only small increases in threshold values. Moreover, these adjustments appeared to be relatively safe, as our data showed only a small increase in alerts requiring clinical actions, implying limited clinical consequences. Additionally, since the start of the program, we have not experienced any adverse events like emergency department admissions or major adverse cardiovascular events. However, in general, triggering simple alerts can still be relevant to some degree, as one-off extreme health values should always be assessed on an individual basis, and general safety reasons can sometimes justify a larger number of alerts. Alert threshold adjustments should, therefore, always be balanced carefully with clinical risks, and any adjustment made should be reassessed periodically to ensure no clinically important events are missed.

### A Framework for Alert Reduction and Workflow Optimization

**Figure 3.**
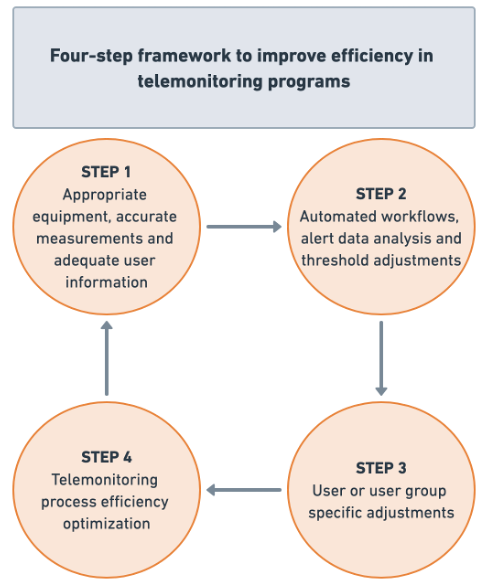
Four-step framework to improve efficiency in telemonitoring programs.

We propose the following alert reduction and telemonitoring process optimization framework for proactive care path embedded telemonitoring programs. This framework (Figure 3), based on 4 distinct steps, outlines a systematic approach to optimize efficiency and can be used for the design, implementation, and optimization of care path-embedded telemonitoring programs.

#### Step 1: Appropriate Equipment, Accurate Measurements, and Adequate User Information

To ensure reliable and adequate measurements, users need to be provided with reliable and validated equipment [10]. Use new relevant medical device features if applicable. Additionally, confirm that the user has received all the necessary measurement instructions and confirm that the users can perform an adequate measurement (for example, a resting “test” BP measurement). Provide users with the correct information during technical issues and educate users on actions to take during acute health issues [10]. Inform users if any relevant changes are made in the remote monitoring program (eg, changes in measurement schedules or new functionalities). Periodically provide personalized feedback to users to ensure long-term reliable measurements, prevent inactivity, and improve overall adherence to the telemonitoring program [25]. Provide users with relevant disease- or lifestyle-specific information to enhance health literary and disease insight, which can both reduce the overall alert burden.

#### Step 2: Automated Workflows, Alert Data Analysis, and Threshold Adjustments

Use automated workflows within the telemonitoring program to improve efficiency. These workflows can, for example, trigger automated messages (eg, a message requesting to repeat a certain measurement) following off-target measurements or process alerts independently. This empowers users to partly take responsibility themselves in case of off-target measurements, which can facilitate telemonitoring in large population groups.

Analyze all generated alerts in terms of their origin and conduct a periodic scenario analysis [21] to assess the impact of threshold adjustments in terms of alert reduction and clinical action requirements [21]. These adjustments should be balanced with clinical risks prior to final adjustment and should always be re-evaluated [23]. Additionally, potentially relevant clinical events after threshold adjustment should always be monitored. Data analysis and algorithm optimization can be performed internally but can also be based on new scientific evidence or guidelines. Remove any measurements from the telemonitoring algorithm that have no clear clinical benefit or consequences. Make use of complex alerts [13,19,20] and clinical algorithms [19,22,24] to filter alerts in terms of clinical relevance, to provide clinical decision support based on measurement trends, and to prevent clinical decision-making on single off-target measurements (“simple alerts”).

#### Step 3: User or User Group Specific Adjustments

Periodically identify users or user groups that generate a large proportion of the overall alert burden in the telemonitoring program (eg, the 10 users or a specific subgroup of users triggering the most alerts, depending on the overall number of users participating in the telemonitoring program). Discuss these users or user groups in a multidisciplinary setting with both the responsible health care provider and the telemonitoring staff involved. Investigate specific reasons for alert triggers in these users or user groups [10]. Consider temporarily user-specific alert thresholds or use personalized measurement schedules if applicable [26]. Recognize the limitations and difficulties of adhering to or using telemonitoring in specific user groups and engage and involve caregivers as needed.

#### Step 4: Telemonitoring Process Efficiency Optimization

Periodically evaluate the complete clinical workflow and telemonitoring process for potential efficiency improvements [12]. Identify alerts that can be processed independently by a telemonitoring center, for example. Use automated alerts and messages (specific alerts that trigger automated advice). For administrative actions, consider using message templates to save time communicating with users. Involve multiple departments (eg, the user information department) to ensure an efficient initial set-up, for example, by giving the user information department the responsibility for the initial device and telemonitoring instructions. Involve users, for example, during periodic focus group interviews to evaluate and optimize the current care pathway from a user perspective.

### Limitations

There are a few important limitations to consider when interpreting the findings of our study. First, the data analysis is conducted with data from a single-center, single chronic disease (hypertension) telemonitoring program, which affects generalizability. However, we tried to overcome this by making general interpretations during telemonitoring programs for various diseases during framework development. Second, we have currently not incorporated the potential of artificial intelligence or other complex algorithm optimization strategies with regard to adjusting alerts and threshold values. It is to be expected, however, that this will be available and applicable on relatively short notice and should, therefore, be included in future updates of our framework. Third, given the large number of alerts, we were unable to analyze the complete clinical context of each individual alert, which might influence the alert processing data. Fourth, a narrative literature review was used in this study, which could have an impact on the strength of the results derived from the literature review. We opted for this approach, however, as very limited studies are available that specifically report methods on alert reduction and efficiency improvement in telemonitoring programs. Fifth, the framework was created based on a synthesis between the alert-data analysis and narrative literature review. This could, as no formal qualitative method was used, impact the overall strength of the framework. However, given the absence of any other similar framework in the literature, it could serve as a valuable starting point for further improvement in future studies.

Sixth, the proposed threshold increases of 5 and 10 mmHg in the scenario analysis might be too broad for populations where more strict BP management is required (eg, aortic dissection) and these adjustments should therefore always be made after assessing the specific included population in the telemonitoring program.

Finally, the available data on the described cohort of patients was only limited to telemonitoring data as the data analysis was used to support the proposed framework. The absence of clinical data could impact generalizability of the findings to certain populations (eg, hypertension, heart failure) included in other telemonitoring programs.

### Conclusions

We developed a framework for alert reduction and telemonitoring process optimization in care path embedded telemonitoring programs. The framework includes 4 steps aimed at ensuring accurate measurements, alert, and alert-processing optimization, focusing on individual user needs, and improving overall telemonitoring process efficiency. Future studies should focus on further development of this framework and the evaluation of techniques to enhance efficiency while balancing potential clinical risks.

## Supplementary material

10.2196/66066 Multimedia Appendix 1 Search strategy and results.

10.2196/66066 Multimedia Appendix 2 Telemonitoring algorithms.

## Abbreviations

BP: blood pressure

## References

[1] Baloch S, Baloch MA, Zheng T, Pei X (2020). The coronavirus disease 2019 (COVID-19) pandemic. Tohoku J Exp Med.

[2] Shaver J (2022). The state of telehealth before and after the COVID-19 pandemic. Prim Care.

[3] Parati G, Dolan E, McManus RJ, Omboni S (2018). Home blood pressure telemonitoring in the 21st century. J Clin Hypertens (Greenwich).

[4] Itchhaporia D (2021). The evolution of the quintuple aim: health equity, health outcomes, and the economy. J Am Coll Cardiol.

[5] Azzopardi-Muscat N, Zapata T, Kluge H (2023). Moving from health workforce crisis to health workforce success: the time to act is now. Lancet Reg Health Eur.

[6] Paré G, Jaana M, Sicotte C (2007). Systematic review of home telemonitoring for chronic diseases: the evidence base. J Am Med Inform Assoc.

[7] Huygens MWJ, Voogdt-Pruis HR, Wouters M (2021). The uptake and use of telemonitoring in chronic care between 2014 and 2019: nationwide survey among patients and health care professionals in the Netherlands. J Med Internet Res.

[8] Kruklitis R, Miller M, Valeriano L, Shine S, Opstbaum N, Chestnut V (2022). Applications of remote patient monitoring. Prim Care.

[9] Co Z, Holmgren AJ, Classen DC (2020). The tradeoffs between safety and alert fatigue: Data from a national evaluation of hospital medication-related clinical decision support. J Am Med Inform Assoc.

[10] Radhakrishna K, Bowles K, Zettek-Sumner A (2013). Contributors to frequent telehealth alerts including false alerts for patients with heart failure: a mixed methods exploration. Appl Clin Inform.

[11] Lee NS, Anastos-Wallen R, Chaiyachati KH, Reitz C, Asch DA, Mehta SJ (2022). Clinician decisions after notification of elevated blood pressure measurements from patients in a remote monitoring program. JAMA Netw Open.

[12] Guédon-Moreau L, Finat L, Boulé S (2015). Validation of an organizational management model of remote implantable cardioverter-defibrillator monitoring alerts. Circ Cardiovasc Qual Outcomes.

[R13] Vukovic M Optimization of the alarm-management of a heart failure home-monitoring system. https://cinc.org/archives/2010/pdf/0053.pdf.

[14] World Medical A (2013). World Medical Association Declaration of HelsinkiEthical Principles for Medical Research Involving Human Subjects. J Am Med Assoc.

[15] Vijayananthan A, Nawawi O (2008). The importance of good clinical practice guidelines and its role in clinical trials. Biomed Imaging Interv J.

[16] (2024). Luscii - an omron healthcare service. https://luscii.com/en/home.

[17] Mancia G, Kreutz R, Brunström M (2023). 2023 ESH Guidelines for the management of arterial hypertension The Task Force for the management of arterial hypertension of the European Society of Hypertension: Endorsed by the International Society of Hypertension (ISH) and the European Renal Association (ERA). J Hypertens.

[18] BeterDichtbij (2024). https://www.beterdichtbij.nl.

[19] Cuba Gyllensten IGL, Goode KM, Bonomi AG, Caffarel J, Amft O (2013). Case-load simulation using home telemonitoring data of heart failure patients to assess the impact of new sensor technologies and alerting algorithms on the decision making of healthcare professionals. Int J Integr Care.

[20] Vamos M, Nyolczas N, Bari Z (2018). Refined heart failure detection algorithm for improved clinical reliability of OptiVol alerts in CRT-D recipients. Cardiol J.

[21] Zahradka N, Geoghan S, Watson H, Goldberg E, Wolfberg A, Wilkes M (2022). Assessment of remote vital sign monitoring and alarms in a real-world healthcare at home dataset. Bioengineering (Basel).

[R22] Nguyen P, Schiaffino MK, Zhang Z, Choi HW, Huh-Yoo J (2023). Toward alert triage: scalable qualitative coding framework for analyzing alert notes from the Telehealth Intervention Program for Seniors (TIPS). JAMIA Open.

[R23] Richman T, Fryer M, Titlestad AL, OLeary J, Greaves K, Tung MK (2023). PO-03-059 optimising remote monitoring alert criteria to reduce alert fatigue: an interrupted time series. Heart Rhythm.

[R24] Mazza GL, Dueck AC, Ginos B (2024). Optimization of alert notifications in electronic patient-reported outcome (ePRO) remote symptom monitoring systems (AFT-39). Qual Life Res.

[R25] van Steenkiste J, Verberk-Jonkers I, de Koning S, Voss-de Haan J, de Jong-Verhagen B, Dohmen D (2024). Patient engagement in a hybrid care pathway for hypertension: not one size fits all. J Patient Exp.

[R26] Shalowitz E (2022). Non-Invasive Telemonitoring Programs in Chronic Heart Failure Patients: How Differences in Alert Criteria Affect All-Cause Mortality Outcomes.

