# Supplementary material for: Alert Reduction and Telemonitoring Process Optimization for Improving Efficiency in Remote Patient Monitoring Programs: Framework Development Study
Source: JMIR Med Inform. 2025 Jun 13;13:e66066. doi: 10.2196/66066 (PMC12206671; doi:10.2196/66066)
Supplement: Multimedia Appendix 1 [file medinform-v13-e66066-s001.docx]

| **Database searched** | **Platform** | **Years of coverage** | **Records** | **Records after duplicates removed** |
| --- | --- | --- | --- | --- |
| Medline ALL | Ovid | 1946 - Present | 149 | 146 |
| Embase | Embase.com | 1971 - Present | 186 | 79 |
| Web of Science Core Collection* | Web of Knowledge | 1975 - Present | 17 | 11 |
| Cochrane Central Register of Controlled Trials** | Wiley | 1992 - Present | 21 | 4 |
| Additional Search Engines: Google Scholar*** | | | 57 | 11 |
| **Total** | | | **394** | **251** |

*Science Citation Index Expanded (1975-present) ; Social Sciences Citation Index (1975-present) ; Arts & Humanities Citation Index (1975-present) ; Conference Proceedings Citation Index- Science (1990-present) ; Conference Proceedings Citation Index- Social Science & Humanities (1990-present) ; Emerging Sources Citation Index (2005-present)

***Google Scholar was searched via "Publish or Perish" to download the results in EndNote.

No other database limits were used than those specified in the search strategies

**Embase 186**

(‘home monitoring’/mj/de OR telemonitoring/mj/de OR Telemedicine/mj/exp OR Telehealth/mj/exp OR ‘wearable device’/mj/de OR ‘wearable sensor’/mj/de OR (telemonitoring* OR homemonitoring* OR telemedicin* OR tele-medicin* OR telehealth* OR tele-health* OR telecare* OR ((home OR tele OR remote-patient*) NEAR/3 (monitoring)) OR wearable OR remote-monitor*):ti,kw) **AND** (‘alert fatigue (health care)’/mj/de OR (alert OR alerts OR alarm OR alarms):ti)

**Medline 149**

(*Monitoring, Ambulatory/ OR *Telemedicine/ OR *Wearable Electronic Devices/ OR (telemonitoring* OR homemonitoring* OR telemedicin* OR tele-medicin* OR telehealth* OR tele-health* OR telecare* OR ((home OR tele OR remote-patient*) ADJ3 (monitoring)) OR wearable OR remote-monitor*).ti,kf.) **AND** (*Alert Fatigue, Health Personnel/ OR (alert OR alerts OR alarm OR alarms).ti.)

**Cochrane 21**

((telemonitoring* OR homemonitoring* OR telemedicin* OR tele-medicin* OR telehealth* OR tele-health* OR telecare* OR ((home OR tele OR remote-patient*) NEAR/3 (monitoring)) OR wearable OR remote-monitor*):ti) **AND** ((alert OR alerts OR alarm OR alarms):ti)

**Web of Science 17**

TI=((telemonitoring* OR homemonitoring* OR telemedicin* OR tele-medicin* OR telehealth* OR tele-health* OR telecare* OR ((home OR tele OR remote-patient*) NEAR/2 (monitoring)) OR wearable OR remote-monitor*) **AND** (alert OR alerts OR alarm OR alarms) **AND** (medic* OR health*))

**Google Scholar (only in title) 57**

telemonitoring|”home monitoring”|telehealth|”tele-health”|telecare|wearable|wearables alert|alerts|alarm|alarms medical|health|telehealth|”home monitoring”

telemonitoring|’home monitoring’|telehealth|’tele-health’|telecare|wearable|wearables alert|alerts|alarm|alarms medical|health|telehealth|’home monitoring’
