# Supplementary material for: Alert Reduction and Telemonitoring Process Optimization for Improving Efficiency in Remote Patient Monitoring Programs: Framework Development Study
Source: JMIR Med Inform. 2025 Jun 13;13:e66066. doi: 10.2196/66066 (PMC12206671; doi:10.2196/66066)
Supplement: Multimedia Appendix 2 [file medinform-v13-e66066-s002.docx]

| **Algorithm** | **Measurement frequency** | **Simple alerts** | **Complex alerts** | **Monitoring frequency** |
| --- | --- | --- | --- | --- |
| 180/110 | Daily (twice morning/twice evening) | RR sys >180 of dias >110 RR <100 sys HF <45 HF >120 | 4 or more readings above/below target in last 48 hours (systolic and diastolic blood pressure, heart rate) | Monday to Friday |
| 170/105 | Daily (twice morning/twice evening) | RR sys >180 of dias >110 RR <100 sys HF <45 HF >120 | 4 or more readings above/below target in last 48 hours (systolic and diastolic blood pressure, heart rate) | Monday to Friday |
| 160/100 | One whole week every fortnight (twice morning/twice evening) | RR sys >170 of dias >105 RR <100 sys HF <45 HF >120 | 8 or more readings above/below target in last 7 days (systolic and diastolic blood pressure, heart rate) | Monday to Friday |
| 150/95 | One whole week every fortnight (twice morning/twice evening) | RR sys >170 of dias >105 RR <100 sys HF <45 HF >120 | 8 or more readings above/below target in last 7 days (systolic and diastolic blood pressure, heart rate)  >1-week average BP within range – move to next protocol’ | Monday to Friday |
| 140/90 | One whole week every month (twice morning/twice evening) | RR sys >170 of dias >105 RR <100 sys HF <45 HF >120 | 14 or more readings above/below target in last 7 days (systolic and diastolic blood pressure, heart rate) | Monday to Friday |
| Protocol switch alert (blood pressure on target) |  |  | 1. End of the measurement week: half of all measurements (with at least 14 measurements performed) are within range -> automated switch to next protocol | Not applicable |
